# Symptom modelling can be influenced by psychiatric categories: choices for research domain criteria (RDoC)

**DOI:** 10.1007/s11017-017-9416-x

**Published:** 2017-07-10

**Authors:** Sam Fellowes

**Affiliations:** 1 Department of Politics, Philosophy and Religion, Lancaster University, Lancaster, LA1 4YL UK; 2 Department of Psychology, Lancaster University, Lancaster, LA1 4YF UK

**Keywords:** RDoC, Symptoms, Psychiatric categories, Factor analysis, DSM, Symptom-based approaches

## Abstract

Psychiatric researchers typically assume that the modelling of psychiatric symptoms is not influenced by psychiatric categories; symptoms are modelled and then grouped into a psychiatric category. I highlight this primarily through analysing research domain criteria (RDoC). RDoC’s importance makes it worth scrutinizing, and this assessment also serves as a case study with relevance for other areas of psychiatry. RDoC takes inadequacies of existing psychiatric categories as holding back causal investigation. Consequently, RDoC aims to circumnavigate existing psychiatric categories by directly investigating the causal basis of symptoms. The unique methodological approach of RDoC exploits the supposed lack of influence of psychiatric categories on symptom modelling, taking psychiatric symptoms as the same regardless of which psychiatric category is employed or if no psychiatric category is employed. But this supposition is not always true. I will show how psychiatric categories can influence symptom modelling, whereby identical behaviours can be considered as different symptoms based on an individual’s psychiatric diagnosis. If the modelling of symptoms is influenced by psychiatric categories, then psychiatric categories will still play a role, a situation which RDoC researchers explicitly aim to avoid. I discuss four ways RDoC could address this issue. This issue also has important implications for factor analysis, cluster analysis, modifying psychiatric categories, and symptom based approaches.

## Introduction

Psychiatric researchers typically assume that the modelling of psychiatric symptoms is not influenced by psychiatric categories; symptoms are modelled and then grouped into a psychiatric category. This is the basis of techniques like factor analysis; psychiatrists determine what symptoms are present in a cohort then use mathematical techniques to establish how they cluster together.

On this picture, psychiatric symptoms influence which psychiatric categories are employed (a psychiatric category being a collection of symptoms), but the reverse is not true; the psychiatric category does not influence the modelling of symptoms. Even if historically some symptoms were modelled after the psychiatric category had been formed, psychiatrists still typically believe that those symptoms would be modelled in the same way regardless of which or if any psychiatric category is employed. I will show this idea can be misleading. Sometimes the psychiatric category influences how symptoms are modelled, the same behaviour being considered an instance of a different symptom depending upon which psychiatric category is employed.

Though notions that psychiatric categories do not influence the modelling of symptoms is important for substantial areas of psychiatry, I will highlight its importance and how embedded it is primarily through analysing research domain criteria (RDoC), which is a major, relatively new, project. The unique methodological approach of RDoC, which its advocates believe makes it more promising than typical DSM based research, exploits the supposed lack of influence of psychiatric categories on symptom modelling, taking psychiatric symptoms as the same regardless of which psychiatric category is employed or if no psychiatric category is employed.

The RDoC project seeks to remedy the unsuccessful search for causes of psychiatric categories. It takes the inadequacy of existing psychiatric categories as severely holding back causal investigation [1, p. 28]. The RDoC project hopes to remedy this problem by circumnavigating psychiatric categories [1, p. 35]. Rather than link causes with psychiatric categories, RDoC researchers link causes with symptoms [1, p. 32]. No longer basing causal studies on existing psychiatric categories, RDoC researchers hope causal investigation will not be held back by flawed psychiatric categories [1, p. 35]. RDoC researchers believe ‘that conducting research in a manner not constrained to any particular *DSM* diagnosis is of high priority’ [2, p. 90], aiming to ‘uncouple research questions from DSM-IV diagnostic categories’ [3, p. 295]. RDoC’s central methodology rests upon the notion that symptoms can be modelled without being influenced by psychiatric categories, seeing symptoms as a means to reach causes independent of the negative influence of psychiatric categories. If the modelling of symptoms are influenced by psychiatric categories, then psychiatric categories will still play a role, a situation which RDoC researchers explicitly aim to avoid.

I do not claim psychiatric symptoms influence all psychiatric categories; rather, I aim to raise awareness of this possibility through a detailed example and two shorter examples. Additionally, even if this situation is widespread, my purpose is not to question the legitimacy or worth of RDoC. Rather, I aim to make RDoC researchers aware that symptoms sometimes are modelled on psychiatric categories and to show multiple ways in which RDoC researchers could respond to this issue, highlighting the advantages and disadvantages of each approach. Establishing which psychiatric symptoms are influenced by psychiatric categories and therefore how widespread this issue is will require case specific study.

I will show how two individuals can exhibit identical behaviour yet be assigned two different symptoms based on their DSM psychiatric diagnosis. This can still occur even when a methodology explicitly does not employ DSM psychiatric categories, as in the case of RDoC. I will only provide a single detailed example of how identical behaviour is modelled as different symptoms due to psychiatric categories, seeking only to show that this can occur.

I first outline what the RDoC project aims to do and establish its implicit assumptions. I then show that behaviour and symptoms are two different things and this means the same behaviour can be modelled in different ways, which results in different symptoms. I then show how deciding if behaviour is one symptom or another can be influenced by psychiatric categories. I discuss possible ways in which RDoC could address this issue. Finally, I briefly relate this issue to other areas of psychiatry, specifically, factor analysis, cluster analysis, modifying psychiatric categories, and symptom-based approaches, showing it also has implications in areas of psychiatry other than RDoC.

### RDoC

The RDoC project was started in 2009 by the National Institute for Mental Health (NIMH), which is now reorienting research grants away from DSM based research to RDoC based research [4, p. 813]. The RDoC project aims to establish the causal origins of mental illnesses. This is intended ‘to provide a knowledge base for future revisions of the DSM and ICD’ [5, p. 5]. RDoC researchers generally assume that DSM psychiatric categories are flawed and need replacing. Bruce Cuthbert (who was the director of RDoC and is now acting deputy director of the National Institute for Mental Health) and Thomas Insel (who was the director of the National Institute for Mental Health until late 2015) write:

DSM-IV… exhibits serious shortcomings with respect to validity. These include extensive comorbidity among diagnoses, overspecification of categories, and not Otherwise Specified diagnoses…. [DSM] categories, created over a generation ago when brain science was in its infancy, do not represent current knowledge about genetics, neural circuits and neurotransmitters, and behavior. [6, p. 889]

Concerns about these and related grounds have led many to believe that alternative psychiatric categories need to be developed.

RDoC’s relatively novel claim is that existing psychiatric categories hold back causal investigation.^1^ The concern is that employing existing psychiatric categories to find causes, such as a genetic study of autism, only informs on existing psychiatric categories. ‘The current categories have grown so entrenched that using them as a framework for examining conventional disorder categories is nearly impossible’ [10, p. 930]. Searching for causes of DSM psychiatric categories does little to investigate alternative psychiatric categories not yet formulated. RDoC researchers fear that ‘the inertia of diagnostic orthodoxy exerts a powerful hegemony over any alternative approaches, leaving us with much debate but little data with which to construct a new nosology’ [11, p. 1061]. Many psychiatric categories have very weak connection to causes, such as autism being connected to hundreds of genes of very low effect size [12, p. 233]. Those genes may have much higher effect sizes for alternative psychiatric categories, but they may be concealed by the use of current psychiatric categories for causal investigation. This is also true for investigating environmental and neurological findings. Establishing how a multiplicity of causes with low specificity and low sensitivity relate to existing psychiatric categories reveals very little about how those causes, or different causes, would have much higher sensitivity and specificity for alternative psychiatric categories. That current formulations reveal little about alternative formulations is true for all medical and non-medical sciences. However, this is generally not particularly concerning when currently employed scientific formulations are good. There is currently no widespread concern over most sciences, and non-psychiatric medicine is typically considered less problematic than psychiatry [13, p. 9]. In contrast, psychiatry is currently in a crisis over validity [14, p. 9] because of a general feeling that current psychiatric categories are not good and could be substantially improved. RDoC researchers heavily endorse this concern and hope that their relatively unique approach will improve psychiatry.

The solution for RDoC researchers is simply not to use existing psychiatric categories when conducting causal investigation. ‘RDoC is largely agnostic with respect to contemporary diagnostic classifications…. [I]n order to develop an integrative approach to classification that incorporates neuroscience and behavior, it is necessary to free research from constraint by current diagnostic entities’ [15, p. 637]. Instead of linking causes with psychiatric categories, the intent is to circumnavigate psychiatric categories by linking causes with symptoms. ‘Rather than starting with symptom-based definitions of disorders and working toward their pathophysiology, RDoC inverts this process’ [16, p. 4]. RDoC formulates causal mechanisms and sees which symptoms are exhibited in people with those causal mechanisms rather than establishing if those causal mechanisms are present in currently employed psychiatric categories. ‘An RDoC-based study would begin by first selecting a domain and construct and then identifying the independent variable of interest from any column in the RDoC matrix’ [17, p. 26]. The domains and constructs employed currently by RDoC are the ‘five domains of functioning: negative valence systems, positive valence systems, cognitive systems, systems for social processes, and arousal/regulatory systems. Each domain is further subdivided into the fundamental building blocks of RDoC—the constructs…. For example, the negative valence systems domain contains five constructs: active threat (“fear”), potential threat (“anxiety”), sustained threat, loss, and frustrative non-reward’ [17, p. 24].

An RDoC study picks a construct from a domain and tests one of the ‘eight units of analysis in the RDoC matrix: genes, molecules, cells, circuits, physiology, behavior, self-reports, and paradigms’ [17, p. 24]. A study could recruit individuals who exhibit the construct ‘sustained threat’ and investigate the unit of analysis ‘genes’. For example,

a hypothetical study of fear responding might include as participants all those presenting for treatment at an anxiety disorders clinic, without respect to the particular diagnosis. All participants would receive the usual clinical assessment, including a battery of relevant questionnaire instruments, and would also participate in a functional neuroimaging session in which a variety of fearful stimuli (some tailored to the individual’s presenting problems, some given to all participants) are presented. The independent variable in such a study (established post hoc) would be the formation of two groups on the basis of a median split of amygdala responses to fearful stimuli on the neuroimaging assessment…. [T]he dependent variables could be overall severity of distress on various measures, plus duration of disorder, to establish whether participants who are hyporeactive on a fear challenge show overall higher levels of severity and longer durations than do those who are hyperreactive. [15, p. 636]

RDoC researchers believe this approach will avoid many problems associated with DSM-based research.

A hypothetical example highlights what information this approach might yield. Imagine if testing for a particular causal mechanism in depression and borderline personality disorder resulted in a 5% prevalence in each disorder. Whilst interesting, this result would be much more informative if results showed that those 5% with the causal mechanism also exhibited the ‘low social skills’ symptom, whereas the 95% without the causal mechanism did not exhibit low social skills. This would be especially interesting if these results were replicated across many different psychiatric categories, the causal mechanism always being present when a patient has low social skills and absent otherwise. This statistical information is not evident by simply comparing causes and individual psychiatric categories. To each psychiatric category, this causal mechanism is just another low effect cause. In contrast, by comparing causes and symptoms, this cause is now an extremely high effect cause of low social skills. By not employing current psychiatric categories, ‘the goal is to permit investigators to study basic mechanisms as they cut across traditional diagnostic categories’ [6, p. 989]. Without current psychiatric categories holding back causal investigation, a greater understanding of which causes result in which effects will hopefully be gained. In the short term, ‘research using the RDoC approach will be organized on the basis of the putative mechanisms rather than the conventional diagnostic categories’ [15, p. 635]. After accomplishing this short term goal, the longer term goal of producing new, potentially superior, psychiatric categories becomes possible.

At this point, there is little evidence RDoC will actually succeed in these goals, but if currently employed psychiatric categories are as flawed as many believe, then RDoC researchers should be praised for taking an alternative approach which has an as yet undetermined potential to succeed. However, I will show a methodological limitation with the approach of RDoC.

### What RDoC presupposes

The RDoC project appears to hold two important presuppositions relevant to my argument. The first presupposition is explicit, the second is implicit.

Firstly, RDoC researchers explicitly take symptoms as relatively unproblematic. For example, ‘the concern about the current diagnostic environment has not been so much with the symptoms themselves’ [1, p. 32]. Also, ‘classifying behavior is relatively straightforward compared with classifying thoughts and feelings, in that behavior can often be observed directly’ [10, p. 933]. Additionally, ‘it is the grouping of symptoms into what have turned out to be overly heterogeneous syndromes that poses the problems for research’ [2, p. 94]. Finally, a ‘genetic variants will not line up with current diagnostic categories…. [U]nbiased parsing of behavioral features might yield a better correspondence to genotype [than existing psychiatric categories]’ [6, pp. 988–989]. In this quote, Cuthbert and Insel assume that DSM categories group behavioural features in a biased way, and place emphasis on removing that bias. RDoC publications show much greater awareness of epistemic issues with modelling psychiatric categories than with modelling psychiatric symptoms.^2^

Secondly, RDoC implicitly presupposes that symptom modelling is not influenced by psychiatric categories. The symptoms are modelled in the same way regardless of which psychiatric category is employed or if no psychiatric category is employed. Whilst this presupposition is widespread within psychiatry, as I later show, this is particularly central for RDoC because RDoC explicitly aims to avoid the negative influence of psychiatric categories. No RDoC publication states that symptom modelling is influenced (or not influenced) by psychiatric categories, but this seems implicit. RDoC researchers explicitly believe that psychiatric categories can negatively influence causal investigation, and they explicitly believe that this negative influence will cease by no longer employing psychiatric categories. RDoC psychiatrists believe linking causes with symptoms rather than with psychiatric categories will avoid the negative influence of psychiatric categories. This then suggests RDoC psychiatrists believe psychiatric categories do not influence symptom modelling. I later show an instance where symptom modelling is influenced by psychiatric categories, thus challenging the implicit assumption of RDoC. I thus show that this implicit assumption is not always warranted through one detailed example, but I do not claim this will be applicable to all symptoms; establishing how widespread this problem is will require study elsewhere. For now, I first explore what symptoms are and how they relate to psychiatric categories.

Unfortunately, within the RDoC literature there is no in depth discussion of the nature of symptoms, leaving the stance of RDoC researchers towards symptoms as ambiguous. Definitions of symptoms within RDoC literature include ‘an abnormality of some degree that can be expressed quantitatively with respect to its deviation from the usual operation of the function(s) attributed to the construct’ [19, p. 1082] and ‘dimensions of observable behavior’ [15, p. 632]. From this, symptoms could be defined as quantitatively described abnormal behaviour. This seems in line with other definitions of symptoms. For example, the psychiatrist Assen Jablensky says, ‘the primary material out of which the diagnostic entities in psychiatry [categories] are constructed consists of patterns of human behaviour [symptoms]’ [7, p. 140], whilst philosopher of psychiatry Dominic Murphy describes symptoms as ‘observable characteristics’ [20, p. 209]. These definitions suggest that symptoms are abnormal behavioural characteristics (I take ‘characteristics’ as interchangeable with patterns, quantities, and dimensions).

There is an important consequence of these definitions, one not explored in the quoted texts. Behaviour and symptoms should be considered two different things. Behaviour should be thought of as an instance of someone acting in a particular way, the act of exhibiting a behavioural instance. In contrast, a symptom should not be thought of as behaviour but as a behavioural tendency—the tendency to exhibit behavioural instances. Under this demarcation, one act of behaviour is a behavioural instance whereas multiple behavioural instances covering a sufficient span of time would be considered a behavioural tendency.

There are multiple reasons for making this demarcation between behavioural instances and behavioural tendencies. Firstly, a behavioural instance covers a single instance whereas a behavioural tendency covers multiple behavioural instances. For example, assigning an individual the ‘low social skills’ behavioural characteristic is a statement that the individual has previously exhibited, and will likely continue to exhibit, behavioural instances of low social skills. An individual exhibits a particular instance of behaviour at a particular moment whereas symptoms refer to multiple instances of behaviour. Secondly, behaviour manifests in multiple ways whereas behavioural tendencies are a more abstract general statement about behaviour. For example, two different manifestations of low social skills will not be identical, each instance involving different words being spoken, yet both count as instances of a more generalised notion of low social skills. Behavioural tendencies are not just multiple identical behavioural instances, but rather, they have a level of abstraction not present in manifestations of behavioural instances. Thirdly, behaviour manifests in particular contexts, responding to various causal factors, more than the causes psychiatrists might specifically attribute to a behavioural tendency. Low social skills have particular biological or psychological causes. However, exactly how those low social skills manifest—the words spoken, the degree and nature of the social misjudgment—will also be influenced by many other causal factors, such as where individuals are, who and how many people they are talking to, why they are talking, their gender, their age, even day or time. A behavioural instance manifests in a context, produced by both those biological and psychological causes attributable to the behavioural tendency and those other causal factors specific to a context. An individual might exhibit much lower social skills talking to their boss compared to talking to their mother, but this does not entail that ‘talking to their boss’ is required for the ‘low social skills’ behavioural tendency. For this reason also, the ‘low social skills’ behavioural tendency has an abstracted generality not present in behavioural instances of the behavioural tendency.

All this means that symptoms should not be seen as behavioural instances, but instead, as behavioural tendencies (or, more specifically, pathological behavioural tendencies). On this view, symptom modelling means taking clinically significant pathological behavioural instances and modelling them into a pathological behavioural tendency. This modelling process requires decisions over what and how many behavioural instances are required to constitute a symptom. Seemingly pathological behaviour can be exhibited without counting as a symptom. An individual can exhibit many instances of low social skills without qualifying for the ‘low social skills’ symptom: perhaps the number of instances is insufficient or perhaps they only occur in specific contexts rather than pervade most social interactions. Deciding how much and which behaviour constitutes a symptom requires a choice over how behavioural instances should be modelled. The above quoted sources may or may not be willing to recognise this distinction between behaviour (as behavioural instances) and symptoms (as pathological behavioural tendencies), but regardless, the distinctions are implicit in those definitions and should be endorsed.

This separation between behaviour and symptoms can result in relatively similar behaviour being modelled very differently. This is most clear over DSM notions of hallucinations. ‘In certain cultures, distress may take the form of hallucinations or pseudo-hallucination and overvalued ideas that may present clinically similar to true psychosis but are normative to the patient’s subgroup’ [21, p. 103]. Clinically similar behaviour is either true psychosis or non-pathological depending on the specific content expressed by the patient. In another example, fear and anxiety likely appear as identical to non-professionals, and the concepts employed by professionals have considerable overlap. Despite this, there exist formalized criteria demarcating fear and anxiety [22, p. 5]. Behaviour which looks identical at one level of analysis is assigned as different symptoms when taking a more detailed analysis. This highlights that symptom formulation requires a choice.

This choice means that there can be a choice over which symptom a particular behaviour is considered an instance of. The process of symptom modelling might result in a behavioural instance being considered a manifestation of one symptom or as a manifestation of a different symptom. There will be many different grounds for making this decision about how to model symptoms from behaviour; I do not offer an account intended as generally applicable to all psychiatry. Rather, I now offer an example in which this modelling process is influenced by the psychiatric category.

### Psychiatric categories influence symptom modelling

In this section, I will first outline how psychiatric categories can influence symptom modelling, and then back up my argument with reference to scientific texts. Imagine two different individuals exhibit literally identical behaviour in a particular situation. Imagine both individuals exhibit anxiety and the environmental cause was an unexpected change (there was a belief of a particular course of events occurring and anxiety resulted when unexpected environmental changes resulted in an alternative course of events occurring). If sufficient in intensity, and if the behaviour occurs with sufficient frequency, then the behaviour likely counts as a manifestation of a symptom. This symptom likely would be modelled as anxiety, a symptom with many diverse causes. Unexpected changes, fear of driving, financial worries, etc. are all causes of anxiety and they all causally influence behavioural manifestations of anxiety. Despite this, these causes still result in the same symptom being modelled rather than each cause resulting in different symptoms being modelled. They are just one of many possible causal factors, and each one does not result in a different symptom being modelled.

Which symptoms are modelled, however, can change when a wider range of behaviour is considered, taking into account behaviour other than this anxiety resulting from the unexpected change. Imagine one individual generally acts within boundaries of normality whereas the other individual exhibits many symptoms of autism, sufficient that they have a diagnosis of autism. Autistic individuals are well known to struggle with unexpected changes. An autistic person exhibiting anxiety following an unexpected change would likely be assigned the ‘disliking unexpected changes’ symptom rather than, or as well as, the ‘anxiety’ symptom. The psychiatric category changes a causal factor from being just one of many possible causal factors present in specific manifestations to a causal factor which results in a different symptom being modelled. For the ‘anxiety’ symptom, the unexpected change is one of many possible causes of anxiety, of specific manifestations of anxiety. In contrast, behavioural manifestations of the ‘disliking unexpected changes’ symptom will involve unexpected changes as a cause. A different symptom is modelled by moving a causal factor from being just one of many possible causal factors to being required. This shows that which symptom an individual is considered to manifest can be influenced by the psychiatric category being employed, compromising the central RDoC goal of escaping the influence of psychiatric categories.

A look at psychiatric and psychological texts backs up my claims. Disliking unexpected changes is rarely discussed outside of the literature on autism. The forty-four pages on anxiety in DSM-5 do not mention disliking unexpected changes, whereas they are mentioned in the five pages on autism. The closest notion found in the anxiety literature applicable to non-autistic individuals is intolerance of uncertainty. When an individual is ‘faced with ambiguous situations, the uncertainty schema will be activated and could lead to the perception of difficulties where problems do not really exist, leading to non-reality based worries’ [23, p. 800]. Intolerance of uncertainty is distinct from other aspects of anxiety in that the worry is about uncertain future events [24, p. 68] and perception of threat [25, p. 36]. A factor analysis of publications on intolerance of uncertainty showed that researchers focus on two elements: desire for predictability and feeling stuck over decision-making [26, p. 1205]. This is very different from notions of autistic people reacting strongly to environmental changes, typically changes that are neither feared nor predicted in advance. Notions similar to disliking unexpected changes are present to a limited degree in intolerance of uncertainty. Of the twenty questions on the intolerance of uncertainty scale, two are ‘unforeseen events upset me greatly’ and ‘one should always look ahead so as to avoid surprises’ [23, p. 798]. These are among the highest scoring items [23, p. 798]. Disliking unexpected changes can be present among non-autistic people who exhibit anxiety. However, this is conceptualised as part of intolerance of uncertainty, itself conceptualised as related to worry which is conceptualised as related to anxiety. A non-autistic individual who reports anxiety following unexpected changes would likely be noted as exhibiting ‘anxiety’, ‘worry’, or both. In contrast, the autistic individual would be very likely to be given the ‘disliking unexpected changes’ symptom, whether or not he is also given the ‘anxiety’ symptom. This example shows how a psychiatric category can influence how behaviour is modelled into a symptom.

I have shown a detailed instance where a psychiatric category plays a role in determining which symptom an individual has. I do not claim this situation is applicable to every psychiatric symptom (it may be common or rare), but it is unlikely that the situation I describe is an isolated instance. Establishing where this situation occurs will require case study with detailed reference to scientific texts, but here, I will briefly suggest two plausible examples. An individual who was exhibiting the behaviour of pulling out his hair might be assigned the symptom of self harming if he were diagnosed as depressed, whereas this might be assigned the symptom of compulsion if diagnosed with obsessive compulsive disorder, or the symptom of ‘recurrent pulling out of one’s hair’ if diagnosed with trichotillomania (a diagnosis often considered part of an OCD spectrum) [21, p. 251]. In another example, a deluded schizophrenic might believe he does not need to eat, which would be the delusions symptom, whereas a non-schizophrenic individual would likely be considered to exhibit the symptom of ‘disturbance in the way in which one’s body weight or shape is experienced’, which is found in the diagnostic criteria of anorexia nervosa [21, p. 339]. All this means that the implicit RDoC assumption, that symptoms are modelled without being influenced by psychiatric categories, is not always the case.

### Choices for RDoC

RDoC aims to circumnavigate psychiatric categories when investigating causes but psychiatric categories can implicitly influence symptom modelling. To see the consequences of this, and to see how RDoC might respond, it needs to be shown exactly how RDoC intends to conduct causal investigation without employing current psychiatric categories.

The results of causal investigation of mental illness are population dependent; different populations have different prevalence of causes. Current causal investigation typically sets populations by psychiatric categories, thus concealing results obtained through using more relevant populations than potentially deeply flawed psychiatric categories. RDoC specifies multiple strategies for picking alternative populations for causal investigation. Cohorts for study ‘might include all patients presenting at a clinic for serious mental illness, irrespective of primary diagnosis’ [11, p. 1062]. Every patient attending the clinic is causally investigated regardless of their diagnosis. More specific populations can be chosen, such as everyone attending a specific type of clinic (e.g., a clinic for anxiety) [16, p. 5], anyone displaying specific symptoms, anyone with specific risk factors, and anyone affected by specific environmental causes [15, p. 635]. RDoC researchers have multiple ways to pick populations for study without employing current psychiatric categories.

Despite all these options, determining which symptoms are exhibited by individuals within a cohort may still implicitly rely upon psychiatric categories. In the example given above, a RDoC psychiatrist would need to decide if each individual has the ‘anxiety’ symptom, the ‘disliking unexpected changes’ symptom, or both symptoms. There is a possibility that RDoC psychiatrists would model symptoms in the manner a non-RDoC psychiatrist would, namely, the non-autistic individuals receive the ‘anxiety’ symptom whereas the autistic individuals receive the ‘disliking unexpected changes’ symptom. RDoC researchers acknowledge that they will use DSM symptoms, writing, ‘candidate research domains identified by this project must derive their clinical relevance from something. Therefore, a priority for RDoC candidate domains is that they can be related to problem behaviors that can be found in the symptom lists that constitute the symptom criteria for conventional mental disorder categories such as those found in the DSM and the ICD’ [15, p. 635].

Therefore, identifying candidates for RDoC studies can be influenced by what DSM symptoms they are considered to have. Two individuals might exhibit the behaviour of ‘disliking unexpected changes’ but each individual could have had that behaviour modelled as a different symptom during their DSM diagnosis. This could determine which participant meets the requirements for inclusion in a specific RDoC study. Additionally, once the participants are chosen, an RDoC study links the assigned symptoms to causes. Unless the RDoC study re-assesses each participant and models their behaviour into symptoms, the symptoms the study considers the participants to exhibit could plausibly be the same as those they were assigned during their earlier DSM diagnosis. Even if each individual were re-assessed, any RDoC psychiatrist who had been previously trained within a DSM framework, or previously had employed the DSM, would likely find that years of training and psychiatric practice has unconsciously predisposed them to model behaviour into symptoms in a manner similar to the DSM.

On all these grounds, the symptoms considered present within the cohort, which will be linked to causes, could be influenced by psychiatric categories. RDoC psychiatrists should be made aware of this possibility, and aim to be alert to the subtle influence of existing psychiatric categories. However, awareness of the situation still leaves the question of which symptoms should be diagnosed. I now show four possible approaches and mention advantages and problems, highlighted with reference to my above example where alternative symptoms are modelled based upon the role given to unexpected changes. I will withhold judgement over which approach RDoC should take.

Firstly, one might assign symptoms based upon the most relevant cause. Since both the non-autistic individual and the autistic individual are causally affected by unexpected changes, they both should receive the ‘disliking unexpected changes’ symptom. Advantageously, this means symptoms are modelled to cover important causes. Problematically, it is unclear how far this approach should be taken. This approach risks inflating the number of symptoms conceptualised by modelling many new types of symptoms on different causes.

Secondly, one might limit how many different types of symptoms are employed, no longer assigning different symptoms based on so many different causes. Since many different factors cause anxiety, there is no need to have a separate symptom of ‘disliking unexpected changes’. Consequently the autistic person is simply diagnosed as having anxiety. Advantageously, the identical behaviour would be modelled as the same symptom. Problematically, this approach loses much of the richness of symptomatology that is present in existing approaches.

Thirdly, one might see symptoms as less mutually exclusive. A particular behaviour could be assigned as both symptoms, the non-autistic individual and the autistic individual getting diagnosed with both anxiety and disliking unexpected changes. Advantageously, this increases the causal information that is present when assigning symptoms. Problematically, assigning more symptoms to each individual reduces the specificity of symptoms. Normally, there is a great variation of symptoms exhibited by individuals within a cohort, each individual exhibiting many different symptoms. However, being less mutually exclusive results in many more individuals in the cohort having general symptoms like anxiety, reducing the specificity of such general symptoms for linking with causes.

Fourthly, one might accept that psychiatric categories can legitimately influence symptom modelling. The non-autistic individual would be assigned ‘anxiety’ and the autistic individual assigned ‘disliking unexpected changes’. Advantageously, unexpected changes rarely cause anxiety in non-autistic individuals (they are quite prominent in intolerance of uncertainty, but that is only one aspect of anxiety), but they do often cause anxiety in autistic people. Therefore, this approach is much more right than wrong—generally, though not always, assigning relevant symptoms. However, under this approach the RDoC goal of not employing psychiatric categories is not fully accomplished since behaviour is modelled as different symptoms based upon the psychiatric category the individual is diagnosed with. This is problematic if RDoC is correct that currently employed psychiatric categories are flawed and hold back research. If autism is a bad psychiatric category, then it is unclear why it should play any role in modelling symptoms. Ideally, any influence of psychiatric categories on symptom modelling would come from good psychiatric categories, but many fear most psychiatric categories are not good.

Of these four approaches, only the fourth would require using psychiatric categories when formulating symptoms. Therefore, RDoC could adopt an approach which avoids using DSM psychiatric categories. However, all four approaches have advantages and disadvantages. It is far from clear that avoiding the fourth approach, and therefore avoiding the use of psychiatric categories, would be best. These approaches need to be debated; otherwise, RDoC risks adopting an approach unconsciously rather than for good reason. These are not purely empirical questions but partly depend upon values. For example, the second approach has the advantage of simplicity but loses detail. Deciding that simplicity or detail is preferable is a value judgement. Additionally, more than one approach can be simultaneously adopted. Implementing each approach might provide different combinations of symptoms. Causes might better link to the symptoms produced by one approach compared to the symptoms produced by another approach. Finally, if RDoC produces few successes at linking causes to symptoms, then changing which approach is implemented would be an option, potentially generating different symptoms for linking with causes.

I have not argued that RDoC is worthless; it may produce extremely valuable results. Rather, I have shown one particular way in which symptom modelling can be more complicated than RDoC portrays. It would be best to recognise this and openly debate the choice. Otherwise RDoC risks unconsciously adopting what may not be the best approach.

I will now briefly apply my argument to three other areas of psychiatry.

### Implications for other research projects

#### Factor analysis and cluster analysis

Factor analysis also implicitly assumes that symptoms are modelled without being influenced by psychiatric categories. Though it takes many forms, the basic idea behind factor analysis is finding patterns within a set of symptoms exhibited by a population. Maurice Lorr claims that ‘When a psychiatrist identifies a syndrome on the basis of observations of a select sample of patients he notes that certain behaviours and signs go together and form a functional unity. Factor analysis is simply a more systematic rigorous procedure…. [The] factors identified would represent the behaviour syndromes which are now established entirely through clinical observation’ [27, p. 5]. The word ‘entirely’ is instructive, resting upon the notion that symptoms are established first and then the factor analysis informs how to categorise symptoms to reach a best fit. Whilst there are many barriers to describing symptoms purely objectively, it is still generally assumed that those symptoms are prior to psychiatric categories. I have suggested this is not always the case, meaning, the modelling of symptoms from which the best fit is established sometimes may have already been influenced by psychiatric categories. This is also applicable to cluster analysis.

#### Modifying psychiatric categories

Generally, psychiatrists assume that modifying psychiatric categories, as can be done between successive editions of the DSM, does not require modification to symptoms. Psychiatrists just modify how those symptoms cluster, a process described as ‘simply rearranging symptom constellations’ [28, p. 11]. On this picture, modifications to the DSM would typically result in the same symptoms being present in future editions, but some symptoms would be listed under different, or new, psychiatric categories. This picture can be mistaken. When symptom modelling is influenced by psychiatric categories, modifying the psychiatric category may ideally also require modifying the symptoms. For example, behaviour which is statistically significant within the population covered by a psychiatric category might not be statistically significant within the new population covered by a modified psychiatric category. A behaviour moving from being statistically significant to statistically insignificant would be a possible reason for psychiatrists to no longer consider it a manifestation of a symptom, thus, resulting in the symptom itself being modified since it now covers different behavioural instances. The grounds employed to decide when behaviour is significant enough to merit inclusion within a symptom involves a level of choice, but it should not be assumed that a behaviour that is significant in one psychiatric category will be significant in another psychiatric category.

#### Symptom-based approaches to psychiatry

Some theorists advise completely abandoning all use of psychiatric categories. For example, Mary Boyle accepts that the people psychiatry calls schizophrenic may hear voices and have confused thinking, but she also thinks that psychiatry should abandon the psychiatric category ‘schizophrenia’. She advises that psychiatry should ‘acknowledge the person’s behavior and experiences and devote enormous energy and resources to trying to understand why these phenomena occur and what variables influence them, but without inferring unsupported concepts like schizophrenia’ [29, p. 166].

However, where symptom modelling has been influenced by psychiatric categories, abandoning all psychiatric categories still likely entails employing symptoms possibly modelled partially based upon existing psychiatric categories. Symptom-based approaches to psychiatry underestimate the difficulty of removing all influence of existing psychiatric categories. Where this situation is applicable, diagnosing just the symptoms without diagnosing psychiatric categories will not fully escape the influence of current psychiatric categories. To the degree that this is the case, there is a substantial limit on symptom-based projects intending to abandon psychiatric categories.

## Conclusion

It is commonly assumed by psychiatrists that modelling psychiatric symptoms is not influenced by psychiatric categories—that symptoms are modelled in the same way regardless of which, or if any, psychiatric category is employed. This is especially evident in implicit assumptions underlying RDoC. The unique approach of intending to circumnavigate psychiatric categories, which makes RDoC seem so methodologically promising, rests upon an implicit assumption that symptom modelling is not influenced by psychiatric categories. I have shown that this is not always the case. RDoC researchers desire to remove all influence of psychiatric categories on causal investigation. However, the symptoms which are linked to causes may have been modelled implicitly through existing psychiatric categories. Existing psychiatric categories may still play a role, undermining the central aim of RDoC. I have also suggested four possible reactions, but I leave it for others to decide which choice is best. I note, however, that each reaction has advantages and disadvantages; even given RDoC researchers’ desire to avoid influence of psychiatric categories, it is unclear that continuing to allow psychiatric categories to play this role is the least preferable of the four options.

Recognising that psychiatric categories can influence symptom modelling also has important implications for many other research programmes in psychiatry, such as factor analysis and cluster analysis, attempts to revise psychiatric categories, and symptom-based approaches to psychopathology. I do not claim that psychiatric categories influence the modelling of all symptoms. I have only provided a single detailed example and two shorter examples; establishing the prevalence of this situation will require further case specific study. Where applicable, this situation will have important implications for numerous areas of psychiatry.

## Footnotes

1 For non-RDoC sources making this claim, see Jablensky [7, p. 142], Poland [8, p. 46], Sullivan [9, p. 257].

2 For RDoC publications, see the RDoC website [18].
